# Book Reviews

**Published:** 1823-04

**Authors:** 


### CRITICAL ANALYSIS
### OF
### ENGLISH AND FOREIGN LITERATURE
### RELATIVE TO THE VARIOUS BRANCHES OF
### Medical Science.

Quæ laudanda forent, et quæ culpanda, vicissim

Illa, prius, cretâ; mox hæc, carbone, *notamus*.—PERSIUS.

### DIVISION I.

### ENGLISH.

ART. I.—*Historical Sketch of the Opinions entertained by Medical Men respecting the Varieties and secondary Occurrence of Small-Pox; with Observations on the Nature and Extent of the Security afforded by Vaccination against Attacks of that Disease.* In a Letter to Sir JAMES M'GRIGOR, Director-General of the Army Medical Department. By JOHN THOMSON, M.D. F.R.S.E. &c. &c. &c.—8vo. pp. 400; Appendix, 43. Longman and Co. 1822.

[Concluded from page 238.]

THE *third period* of Dr. THOMSON comprises what might be termed a history of Vaccination; and the author either quotes from, or alludes to, all the writers of respectability, who have published either separate works on this subject, or cases in the different periodical Journals. It is not our intention to follow him through these details; which, indeed, are rather to be regarded as matter for reference, than as containing any variety of opinions or doctrines capable of being analyzed. There were, indeed, but two opinions:—one, that vaccination, if perfect, was a complete security against small-pox; and the other, that it was not. Much prejudice, much illiberality, and much misrepresentation, are to be found in the early part of this discussion. The object of the writer was, too often, to establish his own preconceived opinions, rather than to discover truth. At length, however, the opinions of the great majority of medical practitioners preponderated in favour of vaccination; and it was even resolved, at one of the principal establishments in this town, that every person who was vaccinated there should receive a certificate of having gone through the cow-pock, and engaging to pay five guineas should they become affected with small-pox at any future period. In 1808, the National Vaccine Establishment was founded, and has, since that period, annually presented a Report on the subject of Vaccination to the Secretary of State for the Home Department. These Reports have been published, and, as might be expected, have been held as documents of the highest authority: a few extracts from them, in the succession of their dates, may be regarded as illustrating, pretty accurately, the progress of opinion on this subject.

1809.—They "have paid diligent attention to the investigation of such *anomalous* cases as have fallen under their notice, and have kept an exact register of every circumstance as it has occurred, by which they hope to render more perfect the knowledge of the subject, and to secure the public from errors and misrepresentations."

1810.—They "have thought it their duty to continue their investigation of the *few anomalous* cases which have fallen under their notice."—"They have to report, that *no case* of failure has occurred in any individual vaccinated by the surgeons of the nine stations since the commencement of this establishment: that the *few instances of failure* submitted from other quarters to the investigation of the Board in the last year, have been asserted without sufficient proof," &c.

1811.—"That, in some instances, the small-pox has affected persons who have been most carefully vaccinated, is sufficiently established."—"The number of instances of small-pox after vaccination, however, is small; and we may fairly presume that, in proportion as improvements are made in the practice, such occurrences will be still more rare."—"Out of the many hundred thousand persons vaccinated, not a single well-authenticated instance has been communicated to them of the occurrence of a *fatal* case of small-pox after vaccination.

1812.—"The surgeons at the nine stations of this metropolis reported to us, the 14th of last January, that they had *no complaint* of any person vaccinated by them having afterwards had the small-pox."

1813.—"The *failures* of vaccination, which *formerly* occasioned so much alarm, are now become extremely rare, from the *improved* methods which have been adopted."

1814.—"From the introduction of vaccination in 1802, to the 28th February, 1814, it is computed that two hundred thousand persons have been vaccinated; and the medical practitioners unanimously declare that *no instance has occurred of small-pox being contracted after regular vaccination.*"

1815.—"In consequence of the decisive measures adopted in Russia, Germany, France, and Italy, the small-pox has become a very rare disease in those countries; and that, by like means, it is no longer known in Ceylon and at the Cape of Good Hope."

1816.—"We feel it our duty to state, that in the town of Ulverston, during the spring of the last year, various instances occurred of patients having had the small-pox after they had been vaccinated: for the most part, however, it was that mitigated form of the disease which marks the previous vaccination; in all the cases it was mild, and in no instance fatal. The occurrence of these failures was confined to a very limited district; and, as such events have since ceased, although great numbers of other individuals, previously vaccinated, were exposed to the variolous contagion, we are of opinion that the failures were owing to the employment of lymph in succession from a vesicle which had not gone through its stages with perfect regularity; and that even the imperfect vaccine vesicle thus produced has very generally the power of rendering the human frame susceptible of none but the mitigated form of small-pox. A few cases of similar failures have been reported; and most of these in London have been visited by the director of this establishment, *who states them to have arisen from imperfect vaccination.*"

1817.—"The continued investigation of the *failures* of vaccination which have taken place here, lead also to conclusions similar to those of the directors of the Dublin Institution; and it has been found that almost all the subjects of these cases have been vaccinated by *methods less effectual* than those which have been adopted and inculcated by the Establishment; the great success of the practice of which, since its foundation in 1808, is the strongest inducement for the plan being generally followed. For which reason the Board printed a new and corrected edition of their instructions, which contain the practice of the Establishment; and they are now distributing the copies gratuitously over the whole empire. Should these be accurately followed, and every person vaccinated be thoroughly infected with the regular vaccine, the Board are fully convinced that failures would become so rare, as hardly to merit the public attention."

1818.—"In several parts of the United Kingdom, particularly near Edinburgh, an *anomalous disease, bearing some resemblance to small-pox*, has appeared in many persons. It has been described by several professional gentlemen of great eminence and experience. From these statements, it appears that this eruption attacked indiscriminately persons who had been previously vaccinated, who had had the small-pox, or who had not gone through either disease."—"In London, some eruptive cases have occurred in persons who had been previously vaccinated: these the Board have had opportunities of examining; and it has been discovered by the directors, that the eruption, in most instances, was the *chicken-pox;* in a few, the mitigated small-pox: and it should not be passed unnoticed, that in all these latter cases vaccination had been performed and conducted in the manner which was *originally* frequently practised, before the adoption of the *superior* method which has been recommended by the Board, and which they have taken much pains to inculcate in their printed directions."

1819.—"We have regarded it as one of our first duties to consider attentively the different cases of small-pox after vaccination, as they have been transmitted to us. We have endeavoured to investigate them, free from the influence of theory, and solely intent on the discovery of truth: and, when we take into our view the immense number of the vaccinated, when compared with the reported failures;—when we reflect on certain peculiarities of constitution, that will exempt some individuals from all common laws;—when we think on the ignorance and carelessness which the vaccinator has but too often betrayed;—when we recollect the mild form which small-pox is reported to have very generally, though not universally, assumed in the vaccinated,—*we cannot hesitate to assert that our conviction of the experiment of universal vaccination is unshaken.*"

1820.—"It is true that we have received accounts, from different parts of the country, of numerous cases of small-pox having occurred after vaccination; and we cannot doubt that the prejudices of the people against this preventive expedient are assignable, (and not altogether unreasonably, perhaps,) to this cause. These cases the Board has been industriously employed in investigating; and, though it appears that many of them rest only on hearsay evidence, and that others seem to have undergone the vaccine process imperfectly, some years since, when it was less well understood, and practised less skilfully, than it ought to be, yet, after every reasonable deduction, we are compelled to allow that too many still remain on undeniable proof to leave any doubt that the pretensions of vaccination to the merit of a perfect and exclusive security in all cases against small-pox, *were admitted at first rather too unreservedly.* * * * We have the most undoubted proofs, from experience, that, where vaccination has been performed perfectly, small-pox occurring after it is almost universally a safe disease; and, though ushered in by severe symptoms, has hardly ever failed to be cut short before it had reached that period at which it becomes dangerous to life. This CONTROLLING power of vaccination must be admitted as next in importance to its *preventive* influence, and surely justifies our high estimation of the value of this great discovery."

This last report appears to us to contain a fair statement of the question; and here we take our stand. If we have been wrong in our estimate of vaccination, it is surely better to acknowledge the error at once, and to inquire what its real powers are. On its first promulgation, it was assumed to be universal, or almost universal, in its effect as an absolute and permanent preventive of small-pox: if, however, these high pretensions originated in enthusiasm, and have not been entirely borne out by experience, let us not, on that account, undervalue the protecting powers it unquestionably possesses; for we agree with the National Vaccine Board, that the controlling power of vaccination must be admitted as next in importance to its preventive influence. Dr. JENNER, however, seems to have retained to the last the same opinions he originally set forth: for, being asked* whether any thing has occurred to shake his confidence in the power of vaccination to exterminate small-pox altogether, provided it be solely and universally propagated?—he replies, "No; every thing to increase it in a higher degree," &c. And again, being asked on what information he grounds his assertion, that small-pox is entirely unknown in many of the continental kingdoms?—he informs us, that it is from sources "both private and public: from the principal authorities and cities in Europe, as well as the British staff in our possessions in Asia." Now, as this opinion is, in point of fact, at variance with innumerable documents, "both private and public," we can only reconcile the contradiction by supposing that Dr. Jenner did not regard the cases described as small-pox to have been really that disease. On this point, however, the mass of evidence is of such authority and weight as, in our judgment, to turn the balance against him.

There are in this metropolis two obstacles to the propagation of vaccination,—viz. the facility with which parents get persons to inoculate their children with small-pox, and their own prejudices against cow-pox. A judgment may be formed of the extent to which these prevail, by reference to some calculations in the Report of the Metropolitan Infirmary, in a preceding Number of this Journal. The number of patients admitted, during the period embraced in the Report, amounts to 8,475; of these, only 3,446 have been vaccinated. Nor do the parents evince any alacrity in availing themselves of the opportunities afforded at the Infirmary of remedying this deficiency; in consequence of which, out of the number above mentioned, 3,145 remain altogether unprotected,—1,884 having had the small-pox. Of those who have been affected with small-pox, 1,360 have had it in the natural form, and 524 have been subjected to it by inoculation. While this practice is continued, it is vain to hope for the general adoption of vaccination. Yet we are convinced, from much intercourse, at two extensive public charities, with the class of people whose prejudices against cow-pox are the strongest, that the proper methods are not adopted to change their opinions. Medical men seem generally afraid of allowing

* See Dr. GREGORY's correspondence with Dr. JENNER, in a former Number.

the possibility of any eruption subsequent to vaccination being small-pox: they insist, even in the clearest cases, that it is not; forgetting that none of the people are so stupid as to believe the word of the *doctor* before their own senses:—thus, finding our assertions partly incorrect, they are naturally led to reject them altogether. It would, we believe, be much better policy to dwell more upon the mitigating influence of vaccination, (for this is the only safe and undisputed ground,) and less upon its powers as an absolute preventive. Let parents be made to contrast the mild forms of the disease which occur after cow-pox, with the malignant cases which occur in the unprotected, and no arguments will be required to impress the lesson. This judicious use of such cases seems to have been made by the physician to the Small-Pox Hospital:—"And it was gratifying to him to perceive that these, so far from deterring from the practice of vaccination, impressed upon the parents its value, and strengthened their faith in its efficacy."*

A very important conclusion, to which we are unavoidably led by this investigation, is, that, whatever power vaccination originally possessed, such power it still retains. Dr. Thomson has shown, on the clearest evidence, that, even from the first introduction of cow-pox, eruptions resembling small-pox have frequently occurred in those who were guarded by the most perfect vaccination. That these eruptions were generally regarded as chicken-pox, until, from their increasing severity during the late epidemics, medical men were compelled to acknowledge many of them to be really small-pox, rendered more mild, or otherwise modified by the previous vaccination. The first attempt in this country to establish a more accurate diagnosis between small-pox and chicken-pox than had been done by Heberden and Willan, was made by Mr. Brown, of Musselburgh. To his suggestions have lately been added, that the disease is not capable of being communicated by inoculation; that the eruption consists of simple vesicles, which are not cellular, as in small-pox; and that a person who has had pure varicella remains as susceptible as before of the cow-pock. We have elsewhere expressed our opinion as to the common origin of the various forms of varioloid eruptions,† and the question has been already discussed in a former Number of this Journal:‡ we do not, therefore, intend to weary our readers by any thing further on this point, but pass on to the conclusion of Dr. Thomson's work. This is devoted to a history of small-pox in other countries during the last twenty years, and contains dis-

* See the Report of the Practice at the Small-Pox Hospital, in our Number for August, 1822.

† *London Med. and Phys. Journal*, July and August, 1820.

‡ *Ibid.* December 1820.

cussions and opinions so similar to those already detailed, that we shall content ourselves with quoting the conclusions at which our author arrives from a review of the opinions of practitioners in other countries besides Great Britain.

"1st. That, from the first introduction of vaccination into these countries, varioloid eruptions have been observed to occur in the vaccinated, sometimes co-existent with, and at other times subsequent to, cow-pock inoculation.

"2d. That these eruptions have, in some countries, particularly in France, been regarded as the genuine product of vaccine virus, and have accordingly had the epithets applied to them of *vaccine*, *vaccinal*, and *vacciniform* eruptions.

"3d. That in those cases in which it seemed to the observers to be doubtful whether the varioloid eruptions, co-existent with or succeeding to vaccination, arose from the infection of cow-pox, of small-pox, or of chicken-pox, they have not unfrequently been termed *anomalous eruptions.*

"4th. That the vaccinal and anomalous eruptions have exhibited appearances similar to those eruptions which, in this country, have been sometimes denominated chicken-pox, and sometimes small-pox modified by vaccination; as is evident from the appellations which they have received, of *vesicular*, *pustular*, and *horny* eruptions.

"5th. That inoculation performed with the matter of these general vaccinal eruptions, has most commonly produced a *local* affection only; but that, in a few instances, a *general* eruption has been produced similar to that from which the matter had been taken.

"6th. That in those countries (Prussia in particular) in which the varioloid eruptions occurring after vaccination have not been supposed to proceed from the action of cow-pox virus, they have been supposed to arise either from the infection of chicken-pox, or from that of small-pox.

"7th. That of those who have supposed that the varioloid eruptions in the vaccinated have proceeded from the contagion of chicken-pox, some (as M'Donald and Heim) have believed this disease to be a modification of small-pox, and others a distinct and specific disease.

"8th. That under the appellation of spurious small-pox have been included, by practitioners, all those various forms of varioloid eruptions which I have described in my former letter, as having occurred during the late variolous epidemic in Edinburgh, in those who had previously gone through either variolation or vaccination; and that, of these forms of varioloid diseases, the mild vesicular eruption, or *new chicken-pox,* has constituted but a small proportion even in the vaccinated, and a still smaller proportion in the unvaccinated.

"9th. That the numerous diagnostic characters between spurious and genuine small-pox, so ingeniously suggested by Heim, are by himself allowed to be difficult in their application to individual cases.

"10th. That the matter of the eruptions considered to be chicken-pox by Heim, and by those who have adopted his diagnostic characters, was capable of producing, and actually has produced, general eruptions.

"11th. That the matter of eruptions, exhibiting in every respect the appearances which occur in small-pox modified by vaccination, has frequently failed in producing a general eruption; though the local pustule excited by it has not unfrequently been accompanied by a febrile action.

"12th. That in all places where vaccination has not been universally adopted, the varioloid eruptions which have been observed to occur in the vaccinated, during the prevalence of variolous epidemics, have been usually regarded by the enemies of vaccination as the genuine product of small-pox contagion, and as proofs of the failure of that practice.

"13th. That the varioloid eruptions which have been observed to occur in the vaccinated, have been represented, by the advocates of vaccination in other countries, as well as in Britain, as the consequences either of imperfect vaccination, or as cases of chicken pox or spurious small-pox.

"14th. That in every country in which small-pox have prevailed epidemically since the introduction of vaccination, spurious small-pox have been observed, and have attacked chiefly the vaccinated, or those who had previously passed through either the natural or inoculated small-pox; and, accordingly, that in every town and village in these kingdoms, in France, Wurtemberg, Holland, America, the West Indies, and the island of Ceylon, in which the late small-pox epidemic has occurred, small-pox and chicken are reported to have co-existed.

"15th. That the vaccine matter which has now been employed for twenty years upon the continent of Europe, in America, and in both the Indies, as well as in this country, does not appear to have suffered any deterioration in its qualities from cutaneous eruptions or other causes, but has continued, during the whole of this period, to communicate to those inoculated with it all the security it has ever done against the attacks of small-pox, and against the dangers of that disease.

"16th. That the vaccine and varioloid eruptions seem to have occurred as frequently in those countries in which the operation of vaccination has been performed with three, four, or more punctures, as in Scotland and America, where that operation is usually performed by making one or two punctures only.

"17th. That the possibility of the occurrence of genuine small-pox after perfect vaccination, which was so long and so generally denied by the Central Committee of Vaccination in France, and by the advocates of that practice in other countries, has at last been admitted to occur in proportions varying from one or two in a hundred, to one in several millions!

"18th. That the epidemical small-pox, which have spread themselves so extensively over the greater part of the globe, since the year 1814, have been observed, in almost every place in which they have occurred, to attack a considerable number of those who had previously passed through small-pox, and have rendered the possibility of the occurrence of secondary small-pox, which was so strenuously denied by the first medical authorities, a point about which there can now no longer exist any just grounds for difference of opinion.

"19th. That while the epidemical small-pox, which have prevailed during the last seven years, have been every where of a most malignant kind, destroying a large proportion of the unvaccinated attacked by them, very few examples have occurred of their having proved fatal in those who had gone through the process of vaccination. And,

"Lastly, That the diminished susceptibility for small-pox contagion,—the general mildness of the disease when it does occur,—and the almost universal security from danger in the vaccinated, must now be considered as the real advantages which have hitherto been derived from the inestimable discovery of Dr. Jenner."

To all of these positions, and particularly to the last, we would call the attention of the profession and the public: they are not the immature speculations of one to whom the subject is new, nor fanciful opinions derived from books alone. Dr. Thomson has given us the result of more minute and extensive research than any other writer has displayed on this subject, both in the closet and at the bed-side of the patient. He informs us that he has seen *eight hundred and thirty-six* cases of small-pox since June 1818: *two hundred and eighty-one* occurred in individuals who had neither had small-pox nor cow-pox, and of these above *one* in *four* died; *seventy-one* had previously passed through small-pox, and of these *two* died; of *four hundred and eighty-four* who had been vaccinated, only *one* died! Such are the results of our author's inquiry; and let no one say it has proved unfavourable to the cause of vaccination: on the contrary, it has shown the only true grounds on which the greatest discovery in the records of medicine can securely rest; while his researches, his opinions, and his authority, have done much to connect the jarring accounts of medical men,—to reconcile their contradictory views, and restore the confidence in vaccination; a confidence which it would be absurd to deny was beginning to be shaken. Dr. Thomson concludes his letter with a Latin quotation complimentary to Sir James Macgrigor,—sentiments in which all who know how justly they are merited must heartily concur.

The Appendix, which contains forty-three closely printed pages, is a letter to Dr. Thomson from Dr. Andrew Smith, hospital assistant, containing an account of cases of secondary small-pox. He quotes twenty-five writers, prior to the year 1720, who have described small-pox as occurring twice, or even oftener, in the same individual. Between 1721 and 1799, sixty-nine writers are referred to, who mention the recurrence of small-pox; among these are to be found the names of some of the most distinguished men of the period, in England, France, Italy, and Germany. From 1799 to the present time, Dr. Smith has alluded to sixty-six practitioners who have mentioned cases of small-pox after small-pox; forming, in this country alone, some hundreds of well-authenticated examples of the recurrence of this disease.

Art. II.—*Practical Observations on the Treatment and Cure of several Varieties of Pulmonary Consumption; and on the Effects of the Vapour of Boiling Tar in that Disease.* By Sir Alexander Crichton, M.D. F.R.S. Physician in ordinary to their Imperial Majesties the Emperor and Dowager Empress of Russia, and to his Royal Highness the Duke of Cambridge; Knight Grand Cross of the second Order of St. Vladimir, Knight of the Red Eagle of Prussia of the second Class, &c.—8vo. pp. xxxvi, 261. Lloyd and Son, London. 1823.

Most of our readers are probably aware that the author of the work before us published, several years ago, "an account of some Experiments made with the Vapour of Boiling Tar in the Cure of Pulmonary Consumption." Since that period, he has employed the same curative means in private practice; but, it would appear, with results, although frequently successful, yet, upon the whole, less satisfactory than in the hospital in Russia. The subject, however, is one of great importance,—interesting to all, and especially so to the practical physician, of whom it may be said to form the daily bread and daily opprobrium: on this account, we feel no apology necessary for calling the attention of our readers to the interesting work of Sir Alexander Crichton. On reflecting upon the subject of pulmonary consumption, it is impossible not to be struck with the contradictory, unscientific, and useless, if not pernicious, remedies which constitute the routine practice of the present day. Some, regarding the changes which take place in the lungs as the result of inflammatory action, think the disease is to be most effectually arrested by blood-letting; while others, alarmed by the debility which constitutes a leading feature in the complaint, endeavour to support the strength by animal food and other forms of nourishing or stimulating diet: one practitioner invariably gives some particular remedy, as digitalis; another as confidently condemns it. There is probably no disease, the pathology of which has improved more within the last few years, —witness the works of Baillie, Bayle, Laennec, and Baron: there is probably none, the treatment of which has improved less. A more convincing proof can hardly be cited to show that, while our knowledge of structural degenerations advances with rapid strides, our knowledge of the means calculated to remove, or even to arrest these, follows at a pace comparatively very slow. Acknowledging, therefore, as we do, the obscurity in which the curative means proper to this complaint are involved, we cannot, nevertheless, give our assent to the opinion of our author, who, admitting the utility of internal remedies in alleviating particular symptoms, continues—" But it seems a strange hope, and strange conduct, to pretend to cure an ulcer in the lungs, whether scrofulous, phlegmonous, or of whatever kind it be, by internal remedies alone, while it is acknowledged that ulcers on other parts of the body require a local application, independently of all internal treatment." Now we would submit that, in the modern practice of surgery, the local application is in many instances a very secondary part of the treatment; and that ulcers, even when apparently unconnected with disorder of the general system, are often most effectually and speedily cured by constitutional means. In pursuing this illustration which the author has suggested, we would be disposed to view the inhalation of medicated gas, or vapour, as bearing the same secondary importance in the cure of phthisis: if, however, it shall be found to merit even this subordinate rank, the medical practitioner will have gained an important auxiliary in the most fatal disease with which he has to combat.

The practice of inhalation is not one of modern date, having been employed by GALEN, and much commended by his follower RHAZES, both of whom employed arsenic and orpiment for this purpose. But the first systematic attempt to introduce this practice seems to have been made by BENNET, in his "Theatrum Tabidorum." This physician describes two different forms of inhalation, under the names of *suffitus* and *halitus:* the former consisting of the vapours extricated by heat alone, principally from resinous substances and gums,—such as storax, frankincense, guaiacum, &c.; the latter were aqueous vapours from various decoctions,—as hyssop, rosemary, sage, &c. MEAD would appear to have placed much confidence in the power of such vapours, as he complains of the neglect into which the practice had fallen among his contemporaries; and our author refers to various other writers, who have mentioned this practice in one or other of its forms.

Sir Alexander does not profess to write a systematic work, or to give any new description of the pathology of the disease; but he takes up, successively, various questions relative to consumption, and discusses them with reference to their practical application. Into the most important of these we shall follow him, although without an attempt to analyze critically the whole of a work, which embraces too many subjects for our contracted limits.

In the first chapter, we find an appeal to professional men, with respect to the possibility "of curing some of the worst varieties of consumption." There are two different methods by which recovery from any disease may be ascertained: first, if a patient labours under symptoms which unequivocally demonstrate the existence of the disease in question, and subsequently recovers; and secondly, if an individual, having died from some other cause, presents, on dissection, such appearances as could only be produced by the previous existence and subsequent removal of the complaint. The possibility of phthisis having been cured, is supposed to have been established by both these methods. Sir Alexander Crichton is satisfied that he has seen persons affected with genuine phthisis pulmonalis cured, "especially from the efficacy of tar-vapour and temperature." M. Laennec has described certain appearances, which he regards as produced by the softening and evacuation of tubercles, followed by cicatrization of the parts. We agree with the latter in regarding his explanation of these appearances as probably correct; and, with the former, we believe that persons labouring under genuine phthisis do occasionally get better: but to what extent this is effected by artificial remedies, further than avoiding to do harm by injudicious interference, we are not prepared to say. Had the word *recovery* stood in the place of *cure*, we should have acquiesced without demur; at the same time, we acknowledge the justice of the author's argument, "as the cases which terminated in a complete recovery, both in St. Petersburgh and Berlin, were considered to be hopeless, until vapour of heated tar was exhibited,—as they were all getting worse under every mode of treatment, until confined in apartments impregnated with this vapour,—as some of the patients had every external mark of a scrofulous constitution, and as several of these patients had every symptom of tubercular phthisis, it might appear to many to be quite superfluous to dispute about the real nature of the cases in which it produced such remarkable good, evidence in medicine being always dubious: but, as the results of these trials show that suspected cases of consumption, which had resisted every other treatment, have yielded to this remedy, they give proof at least of considerable efficacy, and afford great encouragement for proceeding with further trials."—Waving, therefore, our objection, which some may regard as an unnecessary refinement, and assuming that phthisis admits of cure, either from the efforts of nature or interference of art, we proceed to examine the evidence upon which it is asserted that the vapour of tar is efficacious in producing this favourable result. Before, however, we enter upon this examination, we shall detail some circumstances connected with the application of the vapour, of which it is necessary to be aware: these are taken from the first and tenth chapters of the work.

Tar generally contains pyroligneous acid, which, being the more volatile body, is first disengaged, and proves irritating to the lungs. In order to prevent this, the tar ought first to be boiled for a few minutes in the open air, and subcarbonate of potass added, in the proportion of one or two ounces to the pound. The most effectual method is to mix the potass and tar very intimately with the addition of a little water, by which means the acid is neutralized. The tar ought to be as liquid as possible, and to be allowed to "simmer" at a moderate temperature. The proper vapour is invisible, and is breathed with ease: when a white smoke arises, it excites irritation, and the process must be stopt. In private practice, our author generally selects two adjoining rooms, and shuts up any door which opens directly upon a staircase; in cold weather, the windows are made air-tight, by pasting slips of paper on the crevices; all ingress into this apartment takes place through the adjoining one. "The simplest way of charging the apartment with the tar-vapour is to put about a pint, or upwards, of the prepared tar into any flat dish, of iron, copper, or earthen-ware. This is to be placed on a stand, about a foot from the ground, so as to admit a suitable lamp under it." We are cautioned not to place the apparatus too near the patient at first; and advised, in warm weather, to leave it outside the door, the vapour being admitted by a tube passing through it. The construction of our houses in this country is regarded, however, as placing an almost insurmountable bar to the efficacious administration of this remedy; and, in order to try it on a great scale, the expediency of erecting buildings adapted expressly for the purpose is suggested. In these, the circumstances necessary to be kept in view are the following:—"1st. The possibility of charging, to any degree of concentration, all or any of the apartments with tar-vapour; 2d, a mode of ventilation which shall not expose the patient to a current of cold air during our cold seasons, or indeed at any time; 3d, the possibility of having one large ward, or room, always fully charged and of a due temperature, to which patients can resort for exercise; 4th, the means of preserving an equal temperature in every part of the establishment to which the patients are admitted."

There are two methods in which our author would appear to have employed this remedy: viz.—the constant employment of an atmosphere moderately impregnated with tar, or the occasional and interrupted action of one more highly charged with the vapour. Although cases are given in which both of these have succeeded, yet the preference is given to the latter; the atmosphere, when slightly loaded, giving relief, but failing to produce in the lungs those changes necessary to constitute sanatory action. Sometimes the vapour proves so unpleasant as to render its discontinuance unavoidable; and one instance is related in which the smell of tar, even in its natural state, produced immediate syncope.

In the work before us, general directions are given, from the experience of the author, and a general reference made to cases on which this experience is founded, but without details sufficiently minute to enable the reader to judge for himself, in a satisfactory manner, whether he would or would not admit them as examples of *tubercular* consumption. We now give some of the evidence before us.

The wife of a ropemaker, an Englishwoman, aged thirty-eight, of full habit and florid complexion, in consequence of "neglected pneumonia, became affected with hectic fever, colliquative sweats, diarrhœa, great emaciation, dyspnœa, frequent cough, and the expectoration of great quantities of purulent matter." The husband of this woman employed tar obtained from the roots of the white pine, "which he *(the ropemaker)* thought had more effect than the tar from the birch-bark, which is commonly used in Russia." Howbeit, this patient was much relieved within eight days, and ultimately recovered. The next case, that of Prince T———, is one which merits attention. He was twenty-two years old, with a narrow chest and long neck; frequent cough, great dyspnœa, and scanty expectoration of mucus, mixed apparently with pus; to these symptoms were added copious night-sweats and incipient diarrhœa. He recovered; having, as well as the preceding patient, employed an atmosphere constantly, but moderately, charged with the vapour. In the next case, the patient, we are informed, laboured under "all the more decided symptoms of tubercular phthisis," and great hopes were entertained of his recovery; but these were unfortunately disappointed, owing in some degree to hemorrhagy from the lungs, caused by violent sobbing. In October, 1818, a poor woman was admitted, in the early stage of pregnancy, labouring under "frequent cough, with purulent expectoration, pain in her side, great emaciation, and rapidly declining strength. She had colliquative diarrhœa, occasioning about eight watery stools daily. Her pulse was quick; but it could not be said to be a well-marked hectic fever." On the 25th of November, she was discharged cured, having used nothing but the tar-vapour, with the exception of a decoction of polygala senega, with a little ammonia.

These are the only cases we find detailed in this part of the work, but in the Appendix we find seven cases from Hufeland's Journal for 1820. Of these we have already expressed our opinion in our Historical Sketch for January 1823; but, as they are short, we subjoin them, for the same purpose that Sir Alexander has assigned,—viz. "that impartial judges may be able to form their opinions as to the real nature of the cases mentioned."

"Case I.—Charles Botchers, aged eighteen, a shoemaker's apprentice, was born of consumptive parents. One of his sisters was labouring under consumption at the time of his admission. From his earliest years he was short-breathed; and at the age of twelve years he lost the faculty of hearing in his right ear, in consequence of cold. He had also been attacked with erysipelas in the face, which was followed by a cough and expectoration; but both these complaints ceased.

"At the age of seventeen he was seized with a sore throat, followed by diarrhœa. The first of these ailments never left him, and it was soon followed by hæmoptysis.

"Three months after this accident, during a journey from Hamburgh to Berlin, he was attacked, for the second time, with a spitting of blood, which induced him to apply for admission into the Charité.

"His appearance was similar to that of people who are disposed to pulmonary consumption. He was emaciated, had a hectic fever, pain in the throat, and a cough with expectoration. The functions of his intestines were in their ordinary state. A small bleeding from the arm, and an emetic, produced some alleviation.

"Two weeks after his admission, he began the use of the vapour of tar; the hectic fever left him: but the remedy was continued a month and a half longer, when he was so much better as to leave the hospital.

"He returned, however, on the 19th of August, with a cough, pains in the chest, expectoration, and night-sweats. He continued much in this state until the 16th of October, when he was again admitted into the tar-chamber. Here his amendment was so rapid, that in a short period he was again allowed to quit the hospital, at his particular desire. A slight cough, however, still continued.

"Case II.—John Wolkerla, aged forty-eight, was the son of a consumptive father, and had been short-breathed from his childhood.

"In the spring of 1817, he was attacked with coughing and expectoration, streaked with blood. This was followed by nocturnal sweats, and a rapid loss of flesh. The matter expectorated had at times a putrid taste, at other times a saltish one. He could not sleep on the left side, and he had an oppressive pain under the sternum. The functions of his intestines were in their natural state.

"This patient remained in the tar-chamber from the 28th of July to the 29th of August, and, although his fever appeared at times to be increased, nevertheless the general amelioration of his health was complete, and he was at last dismissed quite cured.

"Case III.—Dorothea Schutz, aged twenty-five, not emaciated, and of great nervous sensibility. She was of a pale complexion and delicate make.

"In the month of January, being then in the middle of her pregnancy, she was seized with a violent cough. After her delivery, at the full term, her cough became much worse, and was accompanied with great expectoration; and this was followed by hectic fever.

"She was received into the tar-vapour chamber on the 25th of September, and on the 22d of October the hectic fever had left her; the expectoration was diminished, but the cough continued. Finding herself better, she did not choose to remain in the hospital, and was dismissed.

"Case IV.– Frederica Raven, aged twenty-three, about a month before she applied at the Charité, had been seized with a hoarseness of voice, a cough and expectoration, and all the symptoms of laryngeal phthisis.

"On the 26th of March, 1818, she was admitted a patient of the hospital. Her disease went on increasing until the 3d of June, and that to a degree that her expectoration was puriform and sanguinolent. At this period she was received into the tar-vapour chamber, where she remained until the 22d of October, when, being almost entirely recovered, she desired to leave the hospital.

"Case V.—John Nauman, a shoemaker's apprentice, aged twenty-five, had been labouring two years with a cough. The quantity of matter which he expectorated daily exceeded a quart, and had a most offensive smell. Every symptom of hectic fever was present, and at times he suffered from pains in his breast. Such was his situation when he was admitted into the Charité, on the 16th of September; soon after which he was seized with the itch. But before this occurred he was admitted into the tar-vapour ward, where he continued until the 5th of November, when, being greatly improved in health, he left the hospital, at his particular request.

"Case VI.—John Schukmann, aged forty-nine years, became consumptive after an imperfectly-treated peripneumony. He was admitted on the 20th of July, and continued in the tar-vapour ward till about the middle of November. He got well in this time, so that every symptom of disease left him, and he was dismissed fully cured.

"Case VII.—Fabian Hartung, aged forty-eight years, was admitted into the tar-vapour ward the 25th of September, where he continued until the 18th of November. On his admission, his complaints were great dyspnœa, under which he had laboured more or less for sixteen years; a cough, with expectoration of a thick, heavy, stinking mucus; hectic fever; nocturnal sweats, and impossibility of lying on his right side. All these symptoms left him, and he was dismissed cured.

"Beside the tar-vapour, he took internally the semina phellandrii aquatici, with sugar of milk."

A private letter from Hufeland to the author contains the following passage:—"Experiments with your tar-vapour continue to be made in the Charitè, and also by me in private practice. In *phthisis atonica*, and especially in the *phthisis trachealis*, it has produced the very best effects; but not so in *phthisis florida*. One of my private patients, who fell into a state of phthisis purulenta after peripneumony, and with whom the most efficacious remedies had been tried in vain, began to exhibit signs of recovery on being constantly confined to an apartment charged with tar-vapour; and by its means the cure was at last completed."

It is difficult to avoid imagining that Sir Alexander, in publishing cases, would select those most favourable to his own views: it is natural that he should do so; nor is it an illiberal assumption on our part. Now, although we would fain flatter ourselves that we deserve to be ranked among the "impartial," yet we cannot admit them as satisfactory. In none of the cases is there evidence of the expectoration consisting of broken-down tubercles or pus: in the fourth case, it is called *puriform;* in the fifth, the word *matter* is used indefinitely; in several no character is given; and in the last it is called *mucus.* It will likewise be observed, that Hufeland speaks of the remedy as very doubtful, or, rather, as decidedly inefficacious in phthisis *florida*; and mentions a case of phthisis *purulenta*, which was cured by it, apparently as something extraordinary. We are therefore compelled, after mature reflection, to repeat the opinion formerly given of the cases related by MM. HUFELAND and NEUMANN; which, indeed, appears to be confirmed by the letter from the former to Sir Alexander Crichton, an extract from which is given above.

It is the misfortune of new remedies to be extolled above their merits; and we are certainly not the less disposed to regard the inhalation of tar-vapour as entitled to serious attention, that it is not ushered into the world with any pretensions to specific operation or universal agency. That Sir Alexander Crichton entertains a favourable opinion of the remedy, is matter of course; but that this is not attended with any blind enthusiasm, will appear from the following quotation, which illustrates, at once, the candour of the author, and gives a general summary of his experience on this interesting subject.

"If any benefit is to be expected from medicine in the cure of consumption, it is surely to be expected with more hope of success from those remedies which can be breathed with perfect ease, and brought into contact with the diseased parts, than from medicines which operate only through the medium of the blood, or by sympathy with the stomach. This suggestion, also, ought to encourage the multiplying and varying the trials of such remedies, which is the chief object of my addressing the medical public on this subject. It must however be remarked, that it will require long and repeated experience to ascertain what are all the varieties and stages of consumption which can be relieved by them or by any medicine whatever. The success of the tar-vapour has been in some cases quite remarkable; while in others, which were apparently similar, it did no good, and in some cases it evidently provoked hæmoptoe. In almost all cases where the tar has been duly prepared, its first effects, in relieving the dyspnœa, cough, and pains in the chest, and in diminishing the expectoration, have been remarkable. These effects, on such patients as, from the state of the lungs, were curable, were soon followed by a progressive and general amendment, until a complete cure was produced. But others, who at first were much benefited by the application, began some time after to struggle, as it were, between the good effects of the medicine and the destructive process of the disease; and, from the number and volume of the tubercles, they at last resisted its action altogether."

It is objected, with much apparent justice, that no fair trial of the remedy has been made in this country: indeed, from his long residence in Russia, the author would seem to have overlooked the difficulties which oppose themselves to charging our apartments permanently with any vapour. In northern countries, every egress of air from within, and ingress of air from without, is guarded against with a care unknown in England: and it is here to be observed, that the equality of temperature thus preserved is of itself alone a most important agent in the cure of pulmonary disease. But these are not the only circumstances mentioned by the author as leading to unfavourable results: it appears that, among the comparatively few trials made in this country, some have been upon subjects the most unfit,—viz. those labouring under hæmoptysis, which symptom was expressly pointed out by Sir Alexander, in his first publication, as prohibiting the employment of the tar-vapour. Some have recommended the use of an inhaler; but the exertion required of the lungs in this operation proves an objection to it in cases were these organs are much less impaired than they must necessarily be in phthisis. To have mentioned the circumstance, is a sufficient caution against this experiment. No notice is taken by the author of Dr. Forbes's paper, published in this Journal for October: this omission is easily accounted for, by supposing that it did not fall under the notice of our author at the time his work was in the press. We must beg leave to refer any one interested in this question to the paper above mentioned, in which a fair trial, upon a scale considerably extended, was given to the inhalation of the tar-vapour, under circumstances which do not seem liable to the objections stated by the author against other similar experiments. The result was unfavourable so far as regards tubercular phthisis, but favourable as regards the chronic forms of bronchitis: such likewise appears to us to be the true reading of Hufeland's cases. As, however, the remedy is certainly deserving of further trial, we copy, for the direction of those who may be disposed to benefit by them, the following general precautions of the author.

"In every case it is necessary to begin with a moderate charge of the vapour of boiling tar, and to increase it gradually in strength.

"When the expectoration is copious, when there is no pain or sense of tension in the chest, it almost always affords relief in the act of breathing, even on its first application; but, if there be any inflammation present, or a very scanty and difficult expectoration, with long paroxysms of coughing, it is often a hurtful, and always a doubtful remedy; and therefore, if found to induce any pain, dyspnœa, or dryness of cough, it ought to be desisted from.

"Head-ache and increase of perspiration are common occurrences on the first administration of the vapour; but these are not motives for abandoning its employment, if it affords relief in breathing, or is not attended with any other bad symptom.

"The vapour of the tar, and the necessary confinement to one or two apartments, the temperature of which is well regulated, render the patient very sensible to every impression of cold; and hence it is of great importance that he be confined for months together, during cold weather, to his apartments.

"Upon the slightest appearance of hæmoptysis, the vapour must be avoided.

"In very dry weather, it is useful to have a basin of wetted sand in the apartment, to supply moisture.

"When the cough and other symptoms are relieved by breathing an atmosphere charged with the tar-vapour, it ought to be increased in force twice or thrice a-day for a few hours each time.

"In the chronic tubercular phthisis, it frequently occurs, during the convalescence of the patient, that a new cluster of tubercles become inflamed, either as a natural event in their progress, or from accidental exposure to cold. This is always known by an aggravation of cough, with scanty expectoration. During this state of things, I seldom apply the vapour but in the most diluted state: but, when the tubercular mass is softened, and begins to be expectorated, I again increase the force of the vapour."

The influence of temperature and climate in preventing and curing consumption, are fully considered. It appears that the disease is infinitely less common in Russia than in England, notwithstanding the severity of the northern climate, while scrofula is more prevalent in the former. This is supposed to arise from the circulation on the surface being kept up by the warmth of their fur clothing, and by the uniformly high temperature of their apartments. The author, as might be expected, takes advantage of these facts to advocate the efficacy of similar means in this country. He condemns the custom of sending patients, already advanced in consumption, to distant parts of Europe, exposed to all the discomforts of a long journey, and separation from the consoling attentions of their friends: "consumptive people are still sent to Nice, to the north of Italy, to the south of France, to Naples, to Cintra in Portugal; all which places are destructive to these unfortunate people." Indeed, medical practitioners are becoming aware of their error by daily experience; and the time is probably not far distant when the advantages of well-regulated artificial temperature will be preferred to the cruel alternative of making a long and fatiguing journey—to the grave.

[To be continued.]

### DIVISION II.

### FOREIGN.

ART. III.—*Memoria sullo Scirro e sul Cancro.* (Transactions of the Imperial Institute of Milan, 1822.)

ART. IV.—*Remarks and Practical Results of Observations on Schirrus and Cancer.* By ANTONIO SCARPA, Emeritus Professor and Director of the Faculty of Medicine in the Royal Imperial University of Pavia; Chevalier of the Royal Order of the Iron Crown; Member of the Royal Academy of Sciences at Paris; F.R.S. &c. *Translated from the Italian, with an Appendix, and some Annotations on the Text.* By JAMES BRIGGS, Surgeon to the Public Dispensary, and Assistant Surgeon to the Lock Hospital.—8vo. pp. 61. Cadell, London. 1822.

THE practice of surgery has its stumbling-blocks, as well as that of medicine, and cancer may still justly claim the foremost rank among these opprobria of our art. If every pathological subject had been simplified or illustrated in proportion to the pains bestowed upon it, and to the number and reputation of those authors who, from the remotest antiquity to the present time, have exerted themselves to clear away the obscurities and to solve the difficulties which yet continue to perplex the practitioner, and render the diagnosis a matter of contention,—schirrus and cancer would long since have ceased to be a reproach to the profession, or so fertile a source of emolument to the unprincipled empiric. But the subject is in itself extremely intricate; a long life and a very extended experience will hardly afford to any one man a sufficient number of opportunities of developing the formation, growth, and termination of this disease, so as to arrive at an exact knowledge of the peculiar changes of structure which form the essential characteristic of the true schirrus. Add to this, the number of important questions that present themselves in the course of the inquiry, and which reasoning cannot influence, but which must be the direct deductions of experience and observation, and that of a person whose mind is free from all preconceived theoretical views, and we shall then have some faint idea of the real difficulties of the task. LE DRAN commences his treatise on Cancer with the following remark:* "Ce n'est que dans les observations qu'on peut puiser des connoissances sur la nature du cancer, et sur la manière de le traiter;" and yet it must be confessed, that the manner of making an observation is not of trifling importance; and upon this subject we cannot forbear quoting an extract from an author of our own age and country, which puts this truth in

* *Memoirs de l'Academie Royale de Chirurgie,* tome iii.

a clear light, equally impressive from the truth of the remarks, and the elegance of the language in which they are couched.*

"While we remain unfurnished with authentic standards by which all observations may be examined, it ought not to excite any surprise if the same name be assigned to two complaints, the histories of which are repugnant to each other; or if opposite modes of treatment be directed for diseases that bear a common appellation. To this want of discriminative principles it may be ascribed, that simple facts have been often perplexed by the subtilty of specious distinctions, and that the mind has been bewildered by the minute ramifications of genera and species. It may also be imputed to the same defect, that affinities have been so frequently discovered, and resemblances traced, where nature had impressed an incompatible disparity.

"The clouded, ambiguous, undecisive state of the art of healing, hath been confessed and lamented in every age: it hath often furnished the gay with materials for wit, and infused into the more sober scepticism or disgust. Many different causes have been assigned, on various occasions, for the slow progress and imperfect condition of this important branch of natural science; but, among the several impediments that have been mentioned, I do not recollect that the almost incommunicable nature of medical knowledge hath been noticed by any writer. The term *incommunicable* must here be understood in a qualified sense,—not as implying that the science is in itself mysterious and inaccessible, but that the knowledge of the experienced practitioner must always be transmitted inadequately, and with difficulty, to the mind of the student. * * * For, as language is not rich enough to furnish words that will perfectly denote all the different shades of colour, though their dissimilitude is obvious when presented to the mind, so there is a species of practical knowledge, composed of simple ideas derived from observation, for which no competent terms have yet been contrived, and which no periphrasis can adequately describe."

But, independently of these considerations, it must be conceded that many of the authors themselves, who have written upon this subject, have tended greatly to mislead us in the inquiry, and consequently to retard its progress. The intellectual, as well as the natural world, sometimes presents to us false lights, whose brilliancy attracts, but which lead only to disappointment and error. Substituting some vivid and agreeable fancy for the more sober but laborious research after truth, these men first form an hypothesis plausible and captivating, and afterwards torture all the facts of the case to support their preconceived notions. We allude more especially to Dr. ADAMS' hydatid theory, which, although now fortunately exploded, continued for a long time, by its novelty, and even by its eccentricity, to mislead the profession.

Upon the subject of cancer, indeed, we are almost afraid that

---

* PEARSON'S *Practical Observations on Cancerous Complaints*, (Preface.)

the labours of the last century have thrown, in reality, no additional light: the merit of most of the best modern publications consists rather in clearing away the incumbrances and misconceptions that involve the disease,—in settling some points of practice relative to the period of operation,—in destroying the pretensions of some favourite specific remedies,—in distinguishing those affections most likely to be mistaken for this formidable malady,—and in endeavouring to solve the main question relative to its local or constitutional origin.

The pamphlet before us was originally published in the Memoirs of the Imperial Institute of Milan: its essential doctrine is that of the local origin of schirrus and cancer; and Mr. Briggs has conferred an obligation upon the profession, in translating and publishing the opinions of a man so justly celebrated as Scarpa, and which otherwise would have been inaccessible to the majority of the profession in this country, at least. We shall now, therefore, proceed to the analysis of the work, offering from time to time such remarks as have suggested themselves to our mind from the perusal.

In a short preface, the translator states his belief that the fact of the local origin of cancer is made out in this little work, from a " more satisfactory body of evidence than has ever been yet laid before the public." It appears also to be his conviction, that the search after a specific remedy for the cure of this disease is a vain one; and that, from the facts obtained by the anatomical investigation of the schirrous tumor, no more rational expectation can be entertained of the restitution of a part, the organical structure of which is found to be wholly subverted, any more than of the dispersion of an encysted tumor, or opake lens of the eye, by such means. The great point to determine he, therefore, justly concludes to be, the precise period when the removal of the diseased part may be effected with the fullest assurance of success. The Appendix contains an account of a disease of the breast, usually considered as cancerous, and " which has been introduced, not merely as tending to illustrate the author's opinion upon this subject, but because it seems to throw some light upon a complaint, of which, from its rare occurrence, we possess but very imperfect information, and which, left to itself, appears to have uniformly a fatal tendency." There are also some notes illustrative of the author's opinions.

The pamphlet commences with an enumeration of those points relative to schirrus and cancer, upon which the profession appear to be still divided, and which are these:—1st. Whether schirrus be purely a local, or a general as well as local affection? 2dly. Whether it attacks, without exception every organic texture of the human body? 3dly. Whether there are any certain marks by which it is distinguishable from every other disease which bears an affinity to it? 4th. Whether its malignancy is equal in degree in all parts of the body?" and these questions lead us directly to inquire, lastly, why cancer, especially the "legitimate glandular," so constantly baffles the hand of the operator, and the action of the most powerful caustics or the actual cautery.

In considering these questions, our author does not adhere to the order in which he has stated them, but commences with considering the second point; and he decides that schirrus and cancer never attack primarily the lymphatic system, nor of necessity the absorbent glands: the mucous glands he also considers as exempt from this affection, as well as all the viscera, with the exception of those internal parts which are furnished with an intimate covering of the reflected skin,—as the larynx, œsophagus, stomach, rectum, vagina, and the neck of the uterus; so that he does not admit the indurations of the brain, liver, &c. under the head of cancerous diseases, but as "morbid phenomena of general scrofulous diathesis, or else indurations either referable to previous acute inflammation imperfectly discussed, or produced by slow and long protracted inflammatory action." (p. 5.) In these remarks we entirely concur; and we consider it no small advance to a knowledge of the true nature of this disease, to confine the term strictly to its original meaning: to call a disease schirrus, because *hardness* is its most striking symptom, is likely to prove a fertile source of mistake, and to lead to most unnecessary severity in the treatment. We are quite aware that the liver and other viscera are frequently the seat of the medullary fungus, which Mr. Hey first introduced to the especial notice of the profession, by the name of the Fungus Hæmatodes; but we are convinced that this disease is altogether different in its leading points from true cancer; that it is unphilosophical and mischievous to confound them with each other; and that they present no point of resemblance, either in their external appearance or internal structure, and are only indebted to the relationship that has been supposed to exist between them, to the equally intractable nature and fatal termination of each. We are happy in having these sentiments confirmed by the opinion of so celebrated a man as Scarpa, though several eminent authors of our own country had previously advocated the same opinion.

With regard to the mucous and salivary glands never becoming the seat of cancer, we find that, although this is the general opinion of the best writers, still Mr. Pearson* observes, that he has seen the parotids affected with a true cancerous disease. Boyer asserts the same fact to have occurred in his practice;

---

* *Practical Observations on Cancerous Complaints.*

but, with this single exception, there seems to be no reason to doubt the exemption of the salivary glands in general.

The next point which our author inculcates is this, "that schirrus and cancer never make their appearance before puberty, and seldom before the twenty-fifth year, in either sex." Here we think the period named too early. In the breast and testes, for example, the true schirrus is seldom called into action until that period of life when their respective functions have ceased, or nearly so; for, whoever will take the pains to peruse the almost endless number of cases of tumors taken from the breasts of females under the middle period of life, will find strong reasons for doubting, both from the history of the case and the dissection of the tumors themselves, whether they were not extirpated more in compliance with the fears of the patient, and the vague and uncertain knowledge of the surgeon, than from any real proof of their true nature. It would be invidious, after the above remark, to point out any particular cases; but many such are to be found in most of the publications on cancer, even within the last fifty years.

"Observation and experience, (says M. Scarpa,) teach us that cancer is never formed but in consequence of legitimate schirrus affecting some of the external conglomerate glands, or of rigid porri, or warts, or malignant tubercles of the external or reflected skin, partaking of the nature of schirrus." Among the glands, the mammary is the most frequently subject to the disease; then the parotid, maxillary, and lachrymal glands, and the body of the testicle; for he doubts whether the epididymis is ever the primary seat of schirrus. In the skin, the schirrus presents the appearance either of a hard wart or tubercle, or dark varicose knot; yet, though these three external characters are different, the internal structure of them all bears a close resemblance to the texture of the glandular schirrus, is intersected by whitish lines, and filled with a viscid albuminous fluid.

With regard to the cancer of the uterus, our author speaks confidently, from the result of anatomical and practical facts, that the primordia of cancer of the uterus are to be traced to the ulceration of one or more of these small schirri, which are formed upon the reflection of skin investing the upper part of the vagina, and the orifice and neck of the uterus. Neither is there, he continues, in the whole records of surgery, any well-authenticated fact of cancer of the uterus having originated from any other part of this viscus.

We come now to the most difficult part of our subject, the *diagnosis of cancer;* because it is quite evident that, for all practical purposes, the most efficient of these marks which our author mentions,—namely, the internal texture of these tumors, cannot be any guide in forming our opinion as to the nature of any particular case. Here the attentive consideration of the preceding phenomena of the disease, and all collateral circumstances, can alone enable us to form a correct judgment: it is not one symptom,—it is the whole taken together, and in connexion with each other, that can alone guide us to the truth. M. Scarpa thinks there can be no difficulty in distinguishing schirrus from all encysted tumors, and that by the stony hardness of this latter disease alone. From scrofulous enlargements we must learn to distinguish it by the habit of body, and the known symptoms of scrofulous predisposition: in adults, cicatrices upon the lymphatic glands, which had suppurated in infancy, will sometimes assist us. "The scrofulous taint of the habit is likewise never confined to one of the external conglomerate glands alone; but affects, at the same time, a greater or less number of lymphatic glands in various parts of the body, particularly in the neck, groins, and axillæ." (p. 12.) The smoothness of the tumor, its comparative softness, are also readily distinguishable by the hand of the surgeon; and, lastly, the dull sense of pain which accompanies this swelling, and which gradually increases as it advances in growth. Our author observes, that, in the testes, scrofulous enlargement is *never* isolated, but is always associated with a similar affection of the lumbar and mesenteric glands. This distinction, however apparent in the dead body, it must be evident cannot alone be a sure guide during life, and consequently will not always avert the recommendation of an operation: for our author himself says, that neither the diseased lumbar or mesenteric glands admit of being felt upon the living subject; and it is only after the excision of the testicle that the increased volume of these glands, and the marasmus consequent thereupon, too clearly prove that the nature of the disease had been mistaken. M. Scarpa is careful to distinguish this condition of the testicle from the malignant soft fungus which sometimes attacks that gland. In this latter case the lumbar glands enlarge, in consequence of the irritation of disease: in the scrofulous affection, their swelling is of a common and simultaneous origin. In these cases, our author asks whether tying the spermatic artery in the groin simply, in the manner in which it has been performed by Professor Maunoir for the cure of sarcocele, may not be sufficient, both to arrest the disease of the testicle, as well as the enlargement of the lumbar glands?

We now come to an enumeration of all those marks which, when met with together, leave no doubt as to the nature of the affection. The schirrus is a disease of advanced, or at least of middle age; it attacks those of a sanguineo-bilious temperament, in whom the general vice of the habit bears no suspicion of scrofula. It is a solitary affection. It is excessively hard and indolent; it increases slowly in every direction; its insensibility is preserved, notwithstanding its increase in size, until it degenerates into cancer. Here we must beg leave to dissent: the presence of occasionally lancinating pains, (or *eclairs de douleur*, as they are called by the French,) frequently denote the commencing activity of this affection; and, although the tumor is insensible to the touch, these darting pains will, at intervals, cause much suffering to the patient. Still it is true that the genuine schirrus is not accompanied by constant pain; which, indeed, appears to be inconsistent with its character. It does not uniformly increase in size; for, in the tuberculated schirrus, as soon as the darting pains commence, so far from increasing in size, it shrinks, drawing inward along with it that portion of the skin to which it has formed adhesion.

The above characters sufficiently distinguish the disease from struma,—from tumors of the conglomerate glands, caused by acute inflammation, which has either been neglected or repelled, —as well as from the medullary fungus, which has its origin in the subcutaneous or intermuscular texture; the remarkable elasticity of these tumors is another characteristic.

The above criteria apply to the diagnostic of glandular schirrus; the following observations relate to those warts, or malignant tubercles, of the external reflected skin. "The real nature of these (he says,) is to be judged of from their universal rigidity and hardness; from their being observed to be deprived of their natural integument, which, when they are benign, retains them within certain limits upon the skin; from the unusual size and depth of the base, which seems to extend beyond the substance of the skin; from their being of a yellowish livid, or dark colour, surrounded by a red circle; from their rapid and almost sudden growth; from the intolerable pruritus excited by them; from the fissures that are formed in them, from which there is a slight discharge, at times, of yellowish, sanguinolent, acrid serum, preceded by sudden darting pains." (p. 21.)

It is a great error in practice, says M. Scarpa, to suppose that any hard, chronic, or indolent tumor may, from lapse of time or other circumstances, be changed into cancer. It is certain that any such tumor may degenerate into a sordid, fungous, spreading ulcer; and of which he adduces many instances. Twice our author had an opportunity of examining a supposed open cancer in the breast, which had existed some years. The patient had complained of an uneasy smarting sensation, but never of darting pains; the ichor discharged from them had not the *lixivial odour;* and the general habit of body was such as is usually met with in scrofulous subjects, and had been so from infancy. Such cases have been noticed by Mr. ABERNETHY,

Sir E. Home, Mr. Bell, and indeed by all the writers on this subject: but the disagreement upon this point is not, practically, of much importance, since it is only in the early stages of the disease that the art of surgery can afford any prospect of relief in true schirrous affection.

We now proceed to give some account of our author's observations upon the *structure of the diseased parts*, and which we have postponed until we had collected together all his remarks relative to the diagnosis of the disease.

"In regard to the difference between the internal altered texture of the strumous conglomerate gland and that of schirrus, the following are the results furnished by anatomical investigation. A coloured size-injection, thrown into the arterial vessels of the strumous gland, penetrates it at first very freely, but becomes soon effused into it, in consequence of the flaccidity and friability of the vessels which supply the substance of the gland. When a section is made of the strumous gland, a vascular compact substance presents itself, filled with a glutinous *(albuminous)* fluid, mixed with a granulous, sebaceous, or cretaceous substance. Between the body of the tumor and its exterior involucrum, there are always some vestiges of *coagulable* lymph; and frequently also, in the internal part of it, indubitable proof of its having suffered inflammation, though slight and passive. In the schirrous tumor, on the contrary, the injection, though of the finest kind, never fills but the principal arterial trunks of the diseased gland. The hardness of the substance composing the schirrus is altogether peculiar to this tumor, and cannot be confounded, even by the most inattentive observer, with that of any other kind of chronic glandular enlargement. It has the appearance of softened cartilage, and strongly resembles the mollified substance of the cartilages and ligaments of the joints, in the change observed in the *white* swelling. When the schirrus is divided through the centre, it presents a whitish surface, equally striped with bands still whiter than itself, like rays drawn from the centre to the circumference, or in the form of ramifications. On pressing upon it, a transparent gelatinous (albuminous) fluid issues from it, which, diffusing itself upon the cut surface, renders it in a short time lucid, and as if it were covered by varnish. Lastly, if the struma and schirrus are macerated together in water for a considerable length of time, the struma is found to separate into a soft, spongy, fimbriated mass; while the schirrus retains nearly the peculiar hardness which it possessed before it was subjected to that process. This remarkable difference in the cohesion between the component parts of the schirrus and those of struma, points out the reason why, in some supposed cases of occult cancer, (but which in reality were of a strumous nature,) cavities have been found containing quantities of pure or bloody serum, of two, four, and even six pounds weight, which certainly could never take place in the intimate, hard, tenacious substance of schirrus."

Our author considers it probable that the gelatinous fluid condensed in any of the external conglomerate glands, as well as the *liquamen* which is fixed and coagulated in the intimate texture of warts or tubercles, is as it were a matrix, containing the germ of a peculiar morbific poison, in a latent or inert state, and which only requires the concurrence of particular circumstances to develop its noxious qualities. Whatever obscurity, he goes on to say, there may be with regard to the primary origin of the morbid principles generated in the animal economy, still he argues, from the example of erysipelas, farunculi, the exanthemata, and numerous others, (many of them, in our mind, however, of a very questionable nature,) that germs of disease are actually formed in the general system, which, not being miscible with the blood, are afterwards, by the power of vitality, expelled entirely from it, or driven only to the skin, or else deposited in some of the external emunctories, and there held, for a greater or less time, in a latent and innocuous state. (p. 26.) In conformity with these views, our author inclines to the belief that the schirrus is only the deposition of the seed of a more formidable malady than schirrus itself, and which remains quiescent until, by a concurrence of internal or external causes, it is brought into activity.

Our author is inclined to doubt the existence of a schirrous *diathesis*: 1st, because it is an isolated disease; 2dly, because, if extirpated before it begins to degenerate into cancer, it is radically cured. With regard to predisposition, he observes:

"Another circumstance to be estimated in investigating the primary origin and essence of schirrus, is that of the *predisposition* to this sad affliction. For, of a number of persons of both sexes exposed to the same combination of causes, which in the schools are called excitant *(produtrici)* of schirrus and cancer, the whole are not attacked with this disorder. I see but in all these causes only such as are *occasional;* as, for example, the suppression of the menstrual flux in women, the hæmorrhoidal in men; the disappearance of cutaneous acrimonies, or of the rheumatism, external violence, deep and long-continued grief of mind, the abuse of venery, and the like. And it is right to observe, that these are not even the only occasional causes; for there are numerous examples of women affected by schirrus and cancer, where the menstruation is regular, and of persons in whom it is neither to be attributed to the suppression of habitual sanguineous evacuations, the disappearance of cutaneous acrimonies, of rheumatism, irregularity in living, or long-continued mental distress. The *efficient* cause, then, of this disease is to be traced to no other source than that of internal elaboration, to which every individual is more or less, or in no measure, predisposed, although exposed to the same *occasional* causes. For these reasons, I do not believe it to have been in any manner proved that there are schirri and cancers which are solely produced by external *causes*, to which some writers have been pleased to attribute a character less formidable than that which is met with in the same disease proceeding from internal morbid action: and my assertion is also founded on experience."

The symptoms which announce the development of the *latent* disease, and the commencement of the second stage, are a pruritus and a sense of burning heat within the substance of the schirrous gland, without any alteration in the appearance of the skin that covers it; to these succeed darting pains, which are not increased by making pressure on the tumor. If the gland which has arrived at this stage of the disease be cut into, instead of the hard uniform substance striped with whitish bands, the appearance is that of a hard lardaceous mass, dotted here and there with red points, excavated in many places by cells of various sizes and depths, containing a viscid, cineritious, sanguinolent fluid, of a highly acrid quality.

Our limits do not permit us to follow our experienced author in his vivid and accurate description of the progress and termination of this appalling disease; a description which applies to the most formidable and regular progress of the cancerous ulcer. It must be recollected, however, that sores, the cancerous nature of which cannot be doubted, do not invariably follow this course, nor assumes exactly the appearances here described; and the cases to be found in all modern writers completely prove this fact.

Mr. Pearson has quoted some cases from Wiseman, which, at the period when he published his observations on Cancer, he doubted whether he ought to consider as fair specimens of that disease, but which subsequent experience has induced him to look upon as real instances of cancerous ulceration. The translator of the work before us was authorized by Mr. Pearson to state this alteration in his opinion.

We have now arrived at the practical application of our author's principles; and it ensues, as a necessary consequence of the premises, that the demolition of the schirrus can never be attended with success, unless the operation shall have been executed previously to the development of the latent morbid seed in the substance of the schirrous gland, or malignant wart or tubercle of the skin; that is to say, before the commencement of the lancinating pains, and infection of the lymphatic glands connected with the seat of the occult cancer: and the instances of success which are to be found in Richter, Morgagni, and others, he justly concludes to have been diseases of a totally different nature from the real cancer; and it is upon these grounds that he is disposed to reconcile the very discordant results of the operations recorded by Hill, Benjamin Bell, by the elder Munro, Callisen, Boyer, &c. In the fatal cases the operation was performed too late,—that is, after the morbid leaven had become developed; and in the first-named instances many other diseases were included in, and contributed to, swell the number of fortunate results.

Mr. Pearson, in delivering his opinion upon this practical point, (i.e. upon the proper period of operating,) supposes, says our author, that the schirrus is never, even from its first appearance, confined and circumscribed within the limits of the gland which it occupies, but that the disease extends beyond the sphere of it, and is constantly associated with other smaller schirri, which, from their extreme minuteness, escape observation. By waiting until the principal schirrus has acquired its greatest augmentation, an advantage is obtained of being able to extirpate, together with it, all the surrounding small schirri, which are now become perceptible to the eye and touch of the operator. It will be seen, by referring to Mr. Pearson's work, (p. 31,) that M. Scarpa has in this instance mistaken that gentleman's meaning,—a point which has not escaped the penetration of the translator; and we must therefore, in justice to our eminent countryman, quote the passage in question, in order to show how conformable with reason and truth his opinion is, and how extremely different from the opinion erroneously attributed to him in the pamphlet before us.

"I believe, (says Mr. Pearson,) it is generally true that those morbiferous poisons which have a tendency to perpetuate themselves, taint the constitution more deeply in proportion to the length of time they have acted upon it. But the comparative security from, or danger of, a relapse, can by no means be estimated from the duration of a cancerous complaint. The early extirpation of a cancer confers no peculiar security against the return of the complaint: on the contrary, if the removal of the part were equally complete in two patients, one of whom had been afflicted seven months, and the other seven years, with a cancer, I should esteem the latter patient in less danger of a relapse than the former. My reason for an opinion which to some people may appear singular, is this:—that, when the breast, for example, is affected with cancer, distant parts of the gland may become the seat of the morbid alteration about the same period. These several diseased portions may not advance with an equal celerity; but, while one portion has acquired a considerable bulk, the other altered parts may be scarcely objects of attention. Under such circumstances, the more obviously morbid parts may be removed; but, the disease being only in progression, no man can be certain, without *removing the whole breast*, that he has not left some diseased fibres.* If, however, the disease shall continue without increasing during several years, one may, in general, conclude that its boundaries are more accurately defined."

It is scarcely necessary to say, that this doctrine is in conformity with the sentiments of SHARP, LE DRAN, and all the best authorities, not excepting that of SCARPA himself.

* See some cases of Cancer, by Mr. C. BELL; *Medico-Chir. Trans.* vol. xii.

We shall pass over the quotations from the works of HIPPOCRATES, CELSUS, LUD. MERCATUS, and SEVERINUS, who are all introduced by our author to prove his main point, that the operation can only be undertaken with a prospect of success in the first stage of schirrus; for he employs the term *occult cancer* to denote the second stage, or period when the schirrus has become active. This brings us down to the present age; when, after extracting the opinion of Le Dran,* which tends to the same point of practice, he presents us with the result of the experience of a countryman of his own, FLAJANI, who has performed the operation of extirpating a considerable number of *genuine schirri*, within the first months after the appearance of the disease. Of twenty-seven operations, two patients only have experienced a relapse; the remainder have been perfectly and radically cured. Our author confirms this testimony by his own experience. In the whole course of a long practice, he declares that he has only succeeded in the extirpation of three cases of *genuine* schirrus; these being the only three in which he was so fortunate as to perform the operation within the first months from the appearance of the disease, before the unpleasant sense of pruritus and heat had taken place, and, consequently, that of the lancinating pains. The operation, in all instances, was performed by the removal of the whole mammary gland. In the legitimate schirrus of the testicle, he has, however, effected a cure in a much larger proportion of cases; a difference which he attributes to the more frequent opportunity of extirpating the schirrous testicle during the first stage, than that of amputation of the schirrus of the breast, before it has advanced to the state of occult cancer. (p. 47.)

With regard to warts and malignant tubercles of the skin, he is inclined to allow more liberty with respect to the period of operation; it appearing to him, as it did to Le Dran, that these are of a less virulent nature than the glandular schirrus, or even the malignant tubercles of the reflected skin. Experience has, with our author, confirmed the truth of this remark; having been successful in some cases of this kind, in which not only the darting pains had commenced, but where even fissures were formed, from which there was an occasional discharge of corrosive serum. He, of course, unites the wound, and heals by the first intention.

The pamphlet concludes with the case of a Tyrolese gentleman, aged seventy-four, who had suffered for four years from the presence of three warts upon the left cheek, near the ala of the nose; the middle one equal in size to a bean. At the end of the fourth year, these tubercles were attacked with an into-

* *Mem. de l'Acad. de Chirurg.* vol. iii.

lerable pruritus; the largest soon after became fissured, and discharged some drops of a yellowish, acrid, and sometimes bloody serum. The disease extended at this time from below the internal angle of the left eye to the left commissure of the lips. M. Scarpa extirpated the whole of this mass, beginning his incision a little below the internal angle of the left eye, and continuing it along the left side of the nose, and around the left ala of it, stopping at a transverse line drawn below the nose. In a similar manner, a second incision commencing from the superior point, which, diverging outwardly, fell upon the same transverse line: these two incisions formed the superior triangle. Having done this, he, with the scissors, made two cuts through the substance of the upper lip, which were prolonged as far as the junction of the two preceding incisions with the already-mentioned transverse line; so that the wound was of a rhomboid figure, the lower angle terminating at the outer edge of the lip, the upper one being directly in a line with, and a little below, the internal angle of the eye.* The inferior half was united by means of the twisted suture, as in the operation for the hare-lip; and the superior half was brought into contact by means of the interrupted suture, which it was necessary to pass through the cartilaginous substance of the left ala of the nostril. Every thing went on well. The imprudence of the patient for a time hazarded the healing by the first intention; but absolute rest, rigorous diet, and the aq. lith. acet., dissipated in a few days the swelling that had been produced, and checked the suppuration of the punctures left by the pins. The subject of this operation lived several years afterwards in good health, and without the least appearance of relapse.

The Appendix contains an account of a case of a sanguineous tumor of the breast, which occurred in the practice of the translator, but which want of room compels us merely to notice.

---

* This description is accompanied by a Plate in the pamphlet of M. Scarpa.

